# To Practice Medicine in Texas

**Published:** 1897-07

**Authors:** 


To Practice Medicine in Texas.—The Journal receives in nearly every mail letters asking what are the requisites to practice medicine in Texas. Answering so many inquiries becomes a little monotonous and laborious, the more so from the fact that not one writer out of ten is thoughtful enough to enclose a stamp for reply. It is, really, none of our business; and to forestall future inquiries, and to disseminate the information, we publish below the articles from the Penal Code bearing upon the subject.

The law in force, if we may call that a law which recognizes a diploma of any kind as equal to a license to practice, and simply requires it to be recorded, is the act of August 21, 1876. It would have been an effective law had not an old doctor who was in the Senate at the time, gotten in an amendment which de stroyed the bill, to-wit: "obtain a certificate, OR have a diploma from some medical college regularly chartered." That amendment, which was tacked on on the last reading of the bill, utterly destroyed its efficacy, and we have not been able to get a better one since.

It will be seen by reference to the decisions which we quote just following the law, that the judge did not recognize any diploma, but required the defendant to have a certificate. It seems, from a careful reading of the code, that a diploma is the lawful alternative of a certificate, and if a person has a diploma he need not have a certificate, and vice versa; the diploma is its equivalent. I know positively that the great majority of those now practicing medicine in Texas take that view of it; and, having recorded their diploma, they do not pretend to go before any board for examination and certificate; more is the pity; and there is nothing to compel them to do so. In Kennedy's case, to which reference has so often been made in these pages, Kennedy had neither taken out and registered a certificate, nor had his diploma recorded. Hence, the decision of the court was against him—that he was not a lawful practitioner, notwithstanding he held a diploma, was recognized as a member of the State Association, and was a professor in the Texas Medical College, chair of pharmacy.

It appears, then, if one has a diploma from a medical college chartered by State authority, and has that diploma registered in the office of the clerk of the district court, it is discretionary with him to go before a board and obtain a certificate or not, though there is a difference of opinion on this point, and members of these district boards hold that all persons—holding diploma or not—must be examined and take a license. But nowhere in the law is this position borne out; the law simply says a certificate *or* a diploma.

The following is from the Penal Code, relative to the existing law regulating the practice of medicine in Texas, act of 1876:

UNLAWFUL PRACTICE OF MEDICINE.—ART. 438 [396]. If any person shall practice for pay, or as a regular practitioner, medicine in this State in any of its branches or departments, or offer or attempt to practice without first having obtained a certificate of professional qualification from some authorized board of medical examiners, or without having a diploma from some accredited medical college, chartered by the legislature of the State or its authority, in which the same is situated, he shall be punished by fine not less than fifty nor more than five hundred dollars.

ART. 439 [397]. Each patient visited or prescribed for, or each day's offer to practice, shall constitute a separate offense under the preceding article.

ART. 440 [398]. If any person shall hereafter engage in the practice of medicine in any of its branches or departments for pay, or as a regular practitioner, without having first filed for record with the clerk of the district court in the county in which such person may reside or sojourn, a certificate from some authorized board of medical examiners, or a diploma from some accredited medical college, he shall be punished as prescribed in Article 438. * * *

§ 776. DECISIONS.—The old law was inoperative, because it was irreconcilable with the Revised Statutes upon the subject. (French vs. State, 14 Apps., 76.) Before a physician can legally engage in practice his certificate must be furnished to the district clerk of the county of such physician's residence or sojourn. When recorded it protects the practitioner while he resides or sojourns in the county where it is recorded; but if he changes his domicile to another county he must, before engaging in practice there, furnish his certificate to the district clerk of the latter county for record. (Hilliard vs. State, 7 Apps., Revised Statutes, Art. 3787.) If the defendant, at the time he practiced medicine, labored under the mistake that his certificate had been filed for record, and if said mistake did not arise from want of proper care on his part, he would be entitled to be acquitted. (Pettet vs. State, 28 Apps., 240.)

ART. 441 [399]. The provisions of this chapter shall not apply to any person who has been regularly engaged in the general practice of medicine in any of its branches or departments in this State, five consecutive years prior to January 1, 1875; nor to any person who may have legally qualified himself to practice medicine under the provisions of an act entitled "An act to regulate the practice of medicine," passed May 16, 1873; nor to any female who may follow the practice of midwifery strictly as such.

§ 779. EVIDENCE.—Where the defendant held himself out to the community as a physician, proof of a single case in which he practiced may be sufficient to warrant a conviction. The State, in cases of this character, may prove the professional capacity in which defendant held himself out to the public. (Antie vs. State, 6 Apps., 202.)

